# Comparative and Predictive Significance of Serum Leptin Levels in Non-alcoholic Fatty Liver Disease

**DOI:** 10.7759/cureus.57943

**Published:** 2024-04-09

**Authors:** Mehwish Qamar, Abeer Fatima, Ambreen Tauseef, Muhammad I Yousufzai, Ibrahim Liaqat, Qanbar Naqvi

**Affiliations:** 1 Physiology, Islam Medical and Dental College, Sialkot, PAK; 2 Physiology, Services Institute of Medical Sciences, Lahore, PAK; 3 Physiology, Combined Military Hospital Lahore Medical College and Institute of Dentistry, Lahore, PAK; 4 Physiology, Sahara Medical College, Narowal, PAK; 5 Anatomy, Rai Medical College, Sargodha, PAK

**Keywords:** body fat percentage, nafld, homa-ir, insulin resistance, leptin

## Abstract

Background

Non-alcoholic fatty liver disease (NAFLD) has emerged as the single most common chronic non-viral liver disease. The burden of the disease on healthcare-providing services has increased tremendously. Although a liver biopsy is the most authentic laboratory investigation for scoring the disease progression, it is an invasive technique. Researchers are vigorously working to find alternate markers for the scoring purpose. Despite the importance and association of leptin with metabolic syndrome and its related disorders, there have been relatively fewer studies on serum leptin and its association with NAFLD.

Objective

This study aimed to investigate variations in serum leptin levels between subjects with and without fibrosis in NAFLD and to assess the predictive value of serum leptin levels in NAFLD subjects.

Materials and methods

The study comprised 130 NAFLD subjects from two tertiary care hospitals in Lahore along with 86 healthy controls that were age, gender, and BMI matched with the subjects. Based on the NAFLD fibrosis score (NFS), the subjects were divided into two sub-groups, subjects with simple steatosis and those with fibrosis. Fasting serum leptin, glucose, and insulin levels were measured using enzyme-linked immunosorbent assay (ELISA). The Kruskal-Wallis test was applied to find differences between the three groups and Fisher's exact test for categorical comparison. To assess the predictive value of serum leptin for steatosis and fibrosis in NAFLD subjects, receiver operation characteristic (ROC) curve analysis was implemented.

Results

The difference in serum leptin level was statistically highly significant (p-value <0.001), with leptin levels of 10 (17.1) ng/mL among controls, 20.5 (21) ng/mL in simple steatosis, and 21 (28.6) ng/mL in fibrosis. The area under the ROC curve was 0.67 and 0.52 for steatosis and fibrosis, respectively. The cut-off value of 12.2 ng/mL showed 70% sensitivity and 50% specificity for steatosis, while at a threshold of 18 ng/mL, leptin demonstrated 40% sensitivity and specificity for fibrosis.

Conclusion

In conclusion, this study found that serum leptin levels are higher in NAFLD subjects compared to healthy controls, and it is a good independent predictor for the detection of liver steatosis.

## Introduction

Non-alcoholic fatty liver disease (NAFLD) often begins with simple fat accumulation and progresses to hepatic inflammation, culminating in fibrosis and cirrhosis. Among the theories proposed to explain these pathophysiological changes, the 'multi-hit theory' has garnered significant attention from researchers. The initial hit involves insulin resistance (IR), which leads to fat buildup in the liver. Subsequently, oxidative stress, adipokines, and cytokines deliver a second blow to the lipid-filled hepatocytes. The third important and independent risk factor is genetic, specifically variations of the palatine-like phospholipase 3 gene (PNPLA3), which causes excessive pumping of free fatty acids (FFAs) into the hepatic parenchyma [1,2].

Leptin, a 16-kDa peptide hormone, plays a crucial role in suppressing hepatic glucose production and improving insulin sensitivity through the adipo-insular axis. However, in conditions such as hyperlipidemia or obesity, leptin resistance emerges, resulting in hyperinsulinemia. This phenomenon contributes to increased adipogenesis and further elevates leptin production. Leptin is believed to have a dual role in NAFLD. IR and steatosis contribute to the proinflammatory and profibrogenic actions of leptin, which may be attributed to human variants of the Lep-R gene. Additionally, when leptin is released from hepatic stellate cells, it triggers the production of proinflammatory cytokines, collagen α1, and growth factors, leading to inflammation and fibrosis of the hepatocytes [3].

Considering the role of insulin and leptin in the pathophysiology of NAFLD, it is suggested that these variables may hold predictive value in assessing the stages of the disease. However, to date, only a few human studies have explored the association of leptin with NAFLD, and the results have been inconsistent. The progression of liver fibrosis to cirrhosis and even hepatocellular carcinoma poses a significant burden on healthcare services. Therefore, further research into the relationship between insulin, leptin, and NAFLD stages is crucial for improving diagnostic and predictive approaches and ultimately alleviating the healthcare burden associated with advanced liver disease.

## Materials and methods

Study population

This cross-sectional study was carried out at the radiology departments of two tertiary care institutions after being approved by the Ethical Research Committee of Postgraduate Medical Institute (PGMI), Lahore. After giving written informed consent, the study participants were included if they met the selection criteria, aged between 18 and 50 years, with hyper-echoic liver patterns on ultrasonography, and deranged liver function tests (ALT > 30 u/L for males and > 19 u/L for females), which were labelled as NAFLD patients [4]. Several factors can impact the disease and its outcomes, which is why subjects with a history of liver cirrhosis, viral hepatitis, type 1 diabetes mellitus, pregnancy, oral contraceptive pill usage, endocrine disorders, and alcohol consumption (more than 20 g/day for women and 30 g/day for men) were excluded from the study [5].

Biochemical and anthropometric variables

The study gathered demographic and clinical data (e.g., medical history, socioeconomic status, previous history, drug history, vitals, and physical examination), along with anthropometric variables, weight, height, and circumference of the neck, waist, and hip.

The biochemical examination comprised liver function tests (ALT and AST), cholesterol, platelet count, and albumin, which were reported using hospital data. The study measured fasting blood leptin and insulin levels using the AccuDiag TM Leptin ELISA Kit (USA) and Calbiotech Insulin ELISA Kit (USA). Serum fasting glucose was measured using a photoelectric colourimeter (AE-11, Tokyo Erma Optical Works, Ltd., Japan) with a glucose-oxidase kit from Pointe Scientific, Inc. USA.

Formulas and calculations

Body mass index (BMI) was measured using the standard calculation. The formulas used in the study to calculate body fat percentage (% BF) in both genders were those employed by the U.S. Armed Forces [6]. Homeostatic model assessment for insulin resistance (HOMA-IR) was measured in all the participants, while the NAFLD fibrosis score (NFS) was calculated only in the NAFLD subjects [1,7].

\begin{equation} BMI=\frac{weight (kg)}{height^2 (m^2)} \end{equation}

\begin{equation} Body fat percentage for the males= 86.010 \times \log_{10}(waist - neck) - 70.041 \times \log_{10}(height) + 36.76 \end{equation}

\begin{equation} Body fat percentage for females = [163.205 \times \log_{10}(waist + hip - neck)] - [97.684 \times \log_{10}(height)] - 78.387 \end{equation}

\begin{equation} HOMA-IR = \frac{{\text{Glucose (mg/dl)} \times \text{insulin (}\mu\text{U/ml)}}}{405} \end{equation}

\begin{equation} \text{NFS} = -1.675 + 0.037 \times \text{age} + 0.094 \times \text{BMI} + 1.13 \times \text{diabetes} + 0.99 \times \left( \frac{\text{AST}}{\text{ALT}} \right) - 0.013 \times \text{platelet} - 0.66 \times \text{albumin} \end{equation}

Study groups

The study population was further divided into the following groups based on abdominal ultrasound and NFS, which is a non-invasive scoring system utilized to assess the severity of the disease in NAFLD patients 

Healthy control (n = 86): Individuals without hyper-echoic liver margins on ultrasound, serving as a control group for comparison.

Simple steatosis (n = 100): individuals with steatosis but without significant fibrosis when the NFS score was < -1.455.

Steatosis with fibrosis (n = 30): individuals with fatty liver disease accompanied by mild fibrosis when the NFS score was -1.455 to 0.676.

In our study, none of the NAFLD patients had significant fibrosis, that is, an NFS score of > 0.676.

Statistical analysis

For the data analysis, Statistical Product and Service Solutions (SPSS, version 21; IBM SPSS Statistics for Windows, Armonk, NY) was employed. The Shapiro-Wilk test was applied for normality assessment. Descriptive values for continuous and categorical data were presented using median (IQR) and frequencies, respectively. The Kruskal-Wallis test was utilized to compare the variables between the groups followed by the post hoc test. For the categorical comparison, Fisher's exact test was deployed. To find out the predictive value of leptin, the receiver operating characteristic (ROC) curve was used to assess its predictivity for both steatosis and fibrosis in NAFLD.

## Results

Demographic

The results from Table 1 indicate notable trends across the three groups. Among the individuals for the controls, only two (2%) were identified as diabetic, compared to a substantial increase in prevalence among those with steatosis (18, 18%) and even more so among those with fibrosis (15, 50%). Similarly, the prevalence of hypertension showed a significant escalation with the progression of steatosis severity, with 12 (14%) individuals in the control group being hypertensive, rising to 39 (39%) and 13 (43%) in those with steatosis and fibrosis, respectively. Notably, there was a clear association between BMI and the presence of steatosis, with a higher proportion of obese individuals observed as the severity of the disease increased. This association was statistically significant, as reflected by the p-values (< 0.001) provided.

**Table 1:** Demographic characteristics of the subjects under study *p-value < 0.001 was statistically highly significant; **Probability measured using Fisher’s exact test

Variables		Healthy control (n=86)	Simple steatosis (n=100)	Steatosis with fibrosis (n=30)	p-value
Diabetics**		2 (2%)	18 (18%)	15 (50%)	<0.001*
Hypertensive**		12 (14%)	39 (39%)	13 (43%)	<0.001*
Body Mass Index (BMI)**	Normal (≤25)	8 (9%)	9 (9%)	3 (10%)	<0.001*
	Overweight (25.0-29.9)	16 (19%)	24 (24%)	0 (0%)	
	Obese (≥30)	62 (72%)	67 (67%)	27 (90%)	

Biochemical variables

The results from Table 2 illustrate several significant trends across different biochemical variables and their association with NAFLD severity. Leptin levels, indicative of adipose tissue mass, showed a progressive increase from the control group (HC) to simple steatosis (SS) and further to steatosis with fibrosis (SF), with median values of 10, 20.5, and 21 ng/mL, respectively. This escalation was statistically significant (p-value < 0.001), particularly evident in post-hoc comparisons between HC and SS. When stratified by gender, similar trends persisted, with both males and females showing higher leptin levels in the presence of steatosis, supporting the role of adipose tissue in the pathogenesis of fatty liver disease. The glycemic variable, fasting glucose, insulin, and HOMA-IR levels also exhibited a significant increase as disease severity progressed (p-value < 0.001), with the post-hoc analysis showing statistically significant differences across all groups.

**Table 2:** Comparison of biochemical variables among the groups using the Kruskal-Wallis test HC, Healthy control; SS, Simple steatosis; SF, Steatosis with fibrosis; IQR, Interquartile range; HOMA-IR, Homeostatic model assessment for insulin resistance; ALT, Alanine aminotransferase; AST, Aspartate aminotransferase *Statistically highly significant with p-value < 0.001

Variable		Normal reference range of variables	HC median (IQR)	SS median (IQR)	SF median (IQR)	p-value	Post-hoc test
Leptin (ng/mL)	Overall	2.05-11.09 ng/mL	10 (17.1)	20.5 (21)	21 (28.6)	<0.001*	(HC, SS)
	Male	2.05-5.63 ng/mL	3.8 (9.2)	9.6 (12)	10.3 (11.2)	0.002	(HC, SS)
	Female	3.63-11.09 ng/mL	16 (10.8)	25 (18.2)	28.3 (32.9)	<0.001*	(HC, SS), (SS, SF), (HC, SF)
Glucose (mg/dL)		80-110 mg/dL	62.9 (43.4)	73.4 (27.6)	98.1 (84.7)	<0.001*	(HC, SS), (SS, SF), (HC, SF)
Insulin (µu/mL)		<25 µu/mL	7 (6.9)	14.3 (11.1)	23.7 (16)	<0.001*	(HC, SS), (SS, SF), (HC, SF)
HOMA-IR		<2	1.3 (1.2)	2.7 (2.2)	5.4 (6.4)	<0.001*	(HC, SS), (SS, SF), (HC, SF)
ALT (u/L)		>30 u/L & 19 u/L for male & female	18 (8)	53 (22)	57 (82)	<0.001*	(HC, SS), (HC, SF)
AST (u/L)		14-20 u/L & 10-36 u/L for male & female	21 (7)	45 (20)	59 (29)	<0.001*	(HC, SS), (HC, SF)
Cholesterol (mmol/L)		<5.17 mmol/L	1.9 (1.1)	3.9 (1.8)	4.1 (2.9)	<0.001*	(HC, SS), (HC, SF)
Albumin (g/L)		35-55 g/L	42 (7)	45 (5)	40.5 (8)	<0.001*	(HC, SS), (SS, SF)

Liver function tests including ALT and AST demonstrated marked elevations in SS and SF groups compared to controls, indicative of hepatocellular injury and inflammation associated with advanced stages of steatosis. Serum albumin was higher in steatosis compared to the other two groups, potentially indicating decreased albumin formation in fibrosis due to the compromised liver parenchyma. Cholesterol levels were elevated in both steatosis and fibrosis compared to controls, with values of 4.1 (2.9) and 3.9 (1.8) vs. 1.9 (1.1) mmol/L, respectively (Table 2).

Anthropometric variables

The results, as shown in Table 3, reveal significant associations between anthropometric measures and the severity of steatosis across different groups and genders. BMI did not show significant differences across the groups due the the methodology employed in the sample selection where the controls were BMI matched with the NAFLD subjects. However, both overall and in gender-specific analysis, significant differences were observed in waist circumference and body fat percentage across the three groups. The median (IQR) for waist circumference in both genders exceeded the normal range (< 90 cm for males and < 80 cm for females). Body fat percentage (% BF) followed a similar trend as waist circumference, with the greatest values found in females with fibrosis and significant differences across the groups.

**Table 3:** Comparison of anthropometric variables among the groups using the Kruskal-Wallis test HC, Healthy control; SS, Simple steatosis; SF, Steatosis with fibrosis; IQR, Interquartile range; BMI, Body mass index * Statistically highly significant with p-value < 0.001

Variable		Normal reference range of variables	HC median (IQR)	SS median (IQR)	SF median (IQR)	p-value	Post hoc
BMI (kg/m^2^)	Overall	18-24.9 kg/m^2^	25.7 (4.9)	27.4 (5.1)	27.1 (4.9)	0.55	None
	Male		25.7 (3.9)	26.8 (5.5)	25.5 (3.1)	0.82	None
	Female		25.7 (4.9)	28.1 (4.7)	28.2 (4.4)	0.47	None
Waist circumference (cm)	Overall	80-90 cm	91.4 (12.7)	96.5 (10.2)	96.5 (5.1)	<0.001*	(HC, SS), (HC, SF)
	Male	<90 cm	91.4 (7.4)	96.5 (8.9)	96.5 (3.8)	<0.001*	(HC, SS), (HC, SF)
	Female	<80 cm	91.4 (15.2)	96.5 (11)	101.6 (15.2)	<0.001*	(HC, SS), (SS, SF), (HC, SF)
Body fat percentage	Overall	20-36%	22.5 (16.8)	26.8 (16.1)	29.9 (23.9)	0.005	(HC, SS), (HC, SF)
	Male	20-26%	22.2 (6.7)	18 (3.3)	16.8 (10.8)	<0.001*	(HC, SS), (HC, SF)
	Female	30-36%	33.6 (13.7)	38.3 (12)	41.9 (9)	0.001	(HC, SS), (SS, SF), (HC, SF)

ROC curve

**Figure 1:**
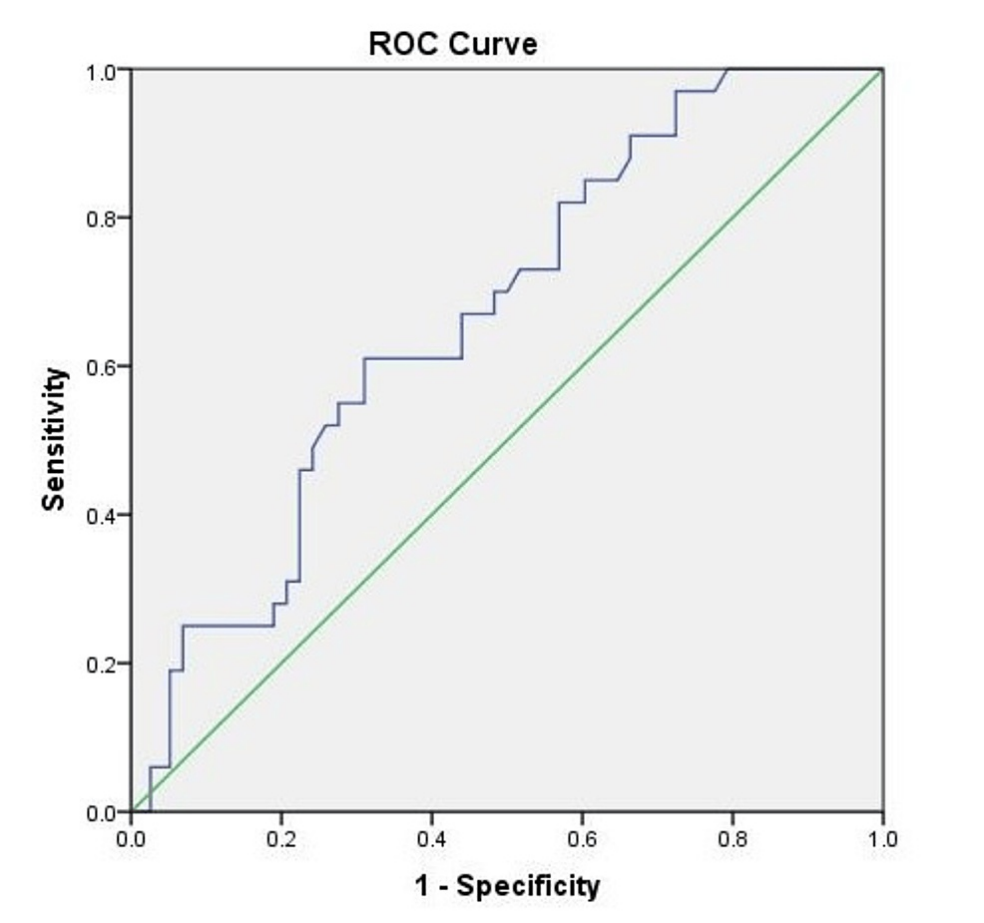
The receiver operating characteristic (ROC) curve depicting the diagnostic accuracy of serum leptin in predicting simple steatosis in NAFLD subjects

Higher levels of serum leptin were discovered as a risk factor for hepatic steatosis using ROC curve analysis, serving as an independent predictor for fat accumulation in the liver parenchyma. At a 95% confidence interval, the area under the ROC curve (AUROC) for discriminating simple steatosis and fibrosis was 0.67, with a p-value < 0.001 (see Figure 1). At a cut-off value of 12.2 ng/mL, leptin predicted simple steatosis with 70% sensitivity and 50% specificity.

**Figure 2:**
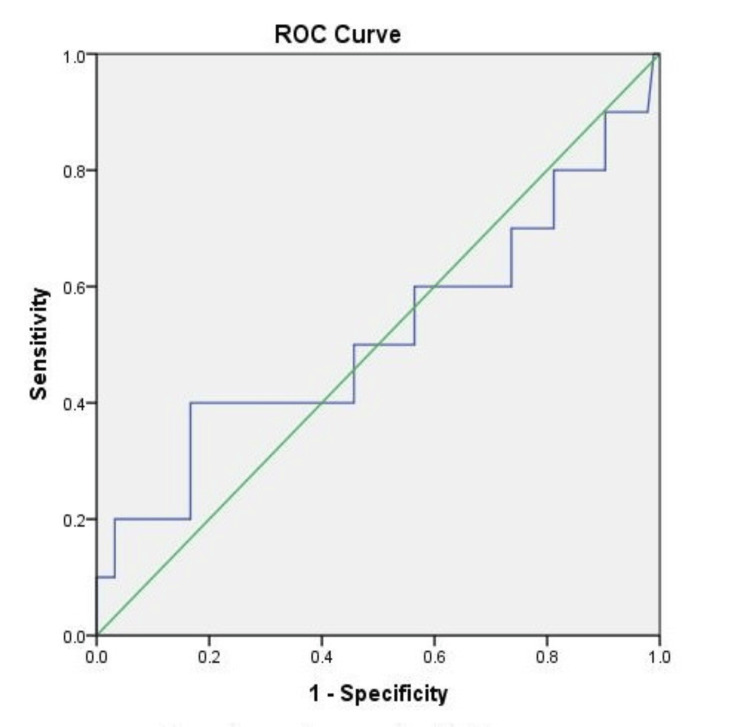
The receiver operating characteristic (ROC) curve depicting the diagnostic accuracy of serum leptin in predicting fibrosis in NAFLD subjects

In contrast, the ROC curve showed that serum leptin cannot detect liver fibrosis (p-value = 0.76), with an AUROC of 0.52 at 95% confidence (Figure 2). At a threshold of 18 ng/mL, leptin showed 40% sensitivity and 40% specificity in predicting fibrosis in NAFLD patients.

## Discussion

This study measured the levels of serum leptin in subjects suffering from NAFLD with and without fibrosis. The patient population selected for the study was stratified into three groups based on fibrosis score. Leptin levels showed a significant difference between the three groups, with the highest levels present in fibrosis. These findings are in line with another study, which showed increased levels of leptin with increasing hepatic fibrosis followed after NAFLD, especially in subjects with high BMI and other features of metabolic syndrome [8]. In the comparison between individual groups, females exhibited a significantly larger difference in serum leptin levels across all groups. This is similar to a study done on data obtained from the United States Third National Health and Nutrition Examination Survey that categorized leptin levels into three groups, noting a higher proportion of women in the highest leptin tertile compared to men when matched for age [9]. This observation may be attributed to the influence of hormones, body fat percentage, and peri-menopausal/menopausal age, factors that potentially elevate the risk of metabolic syndrome and its associated complications, including NAFLD.

In the present study, half of the patients with fibrosis were identified as diabetic or insulin-resistant. Another study, which employed the diabetes liver fibrosis score model to calculate the risk of advanced fibrosis, reported the highest risk in patients with type 2 diabetes [10]. The study identified significant differences in serum levels of glucose and insulin, as well as HOMA IR, exhibiting their highest levels in the fibrotic group. A similar trend was reported in a study suggesting that subjects with hepatic steatosis face a higher risk of developing fibrosis in the presence of insulin resistance compared to its absence [11]. Insulin resistance has been known to accelerate lipolysis in adipose tissue, leading to the development of lipotoxicity and hence inflammation, forming the basis of disease progression to steatohepatitis and liver fibrosis in patients with fatty liver disease [12].

When comparing liver function tests (AST and ALT) and total cholesterol levels, a significant difference was observed among the three groups. This aligns with a study by Marques et al., which reported distinctions in liver function tests between NAFLD and NASH groups even in the presence of similar comorbidities [13]. Serum albumin levels appear to decrease gradually from controls to fibrosis, with a substantial difference between the two groups. This finding is consistent with recent studies that looked at the glucose-to-albumin ratio in people with NAFLD and advanced hepatic fibrosis, revealing a link between a greater glucose-to-albumin ratio and advanced fibrosis, which has a bad prognosis [14,15].

In the current study, hypertension was most prevalent in the fibrotic group. A study on NAFLD subjects across all blood pressure levels found that they had higher blood pressure values with a greater risk of developing advanced fibrosis [16]. The present study showed significantly increased BMI in subjects with fibrosis. These results were in congruence with Schmitz et al. and Loomis et al. who found that the prevalence of NAFLD increases with increased BMI [17,18]. BMI has been regarded as an independent risk factor for the progression of hepatic steatosis to fibrosis [19]. Body fat percentage is significantly higher in the group with fibrosis, especially in females. Body fat percentage and visceral adipose tissue have the strongest association with the severity of hepatic steatosis and fibrosis independent of BMI, as mentioned in a previous study [20].

The optimal cutoff value for serum leptin in predicting the occurrence of steatosis in our study was determined to be 12.2 ng/mL, yielding a sensitivity of 70% and specificity of 50%, with an AUROC of 0.67 and p-value of < 0.001. A study by Manco et al. in 2007 also identified a cutoff value of 14.9 ng/mL as the best predictor for NAFLD with an AUROC of 0.83 [21]. In contrast, Canbakan et al. found that leptin was not an independent predictor for NAFLD [22].

At a threshold of 18 ng/mL, leptin demonstrated a sensitivity and specificity of 40% each in predicting the presence of fibrosis in NAFLD and the curve showed limited discriminatory power when distinguishing between steatosis and fibrosis (AUROC = 0.52 and p-value = 0.76). These findings align with research confirming leptin's involvement in NAFLD but suggest limited prognostic value for hepatic fibrosis [9]. It is worth noting that recent research has emphasized the role of leptin in predicting progressive fibrosis [13].

Limitations and recommendations

There are several limitations to be acknowledged in our study, with the most significant being the absence of a liver biopsy. This technique, although considered the gold standard for assessing liver conditions, is invasive, requires expertise, and is generally not performed in outpatient settings unless there is a clear indication of a significant liver disorder. Nevertheless, the assessment methods utilized in our study are well-validated. Another limitation is the uneven distribution of patients with fibrosis, with a smaller number compared to the other two groups. Additionally, the absence of a substantial number of cases with significant fibrosis poses a concern in drawing definitive conclusions. A study with a larger representation of patients with fibrosis and a more balanced distribution across fibrotic stages would provide a better opportunity to explore the relationship of leptin in the progression of NAFLD. The significant difference in the frequency of known diabetics among the groups is another potential confounding factor that could have influenced the study outcomes in determining the independent predictive nature of leptin. We recommend a prospective approach to investigate leptin and leptin resistance in various stages of NAFLD, considering a more balanced representation of patients and accounting for potential confounders.

## Conclusions

We conclude that a significant difference in leptin levels has been observed among the participants with the highest levels present in the fibrotic group. With good sensitivity and moderate specificity, leptin is a significant predictor for steatosis in the NAFLD spectrum of the disease. However, the predictive value of leptin in fibrosis does not emerge as a robust independent predictor. Its levels appear to be more influenced by gender and general body adiposity rather than the presence of fibrosis in the hepatic tissue.
